# Thermosensitive Shape-Memory Poly(stearyl acrylate-*co*-methoxy poly(ethylene glycol) acrylate) Hydrogels

**DOI:** 10.3390/gels9010054

**Published:** 2023-01-10

**Authors:** Hideaki Tokuyama, Ryo Iriki, Makino Kubota

**Affiliations:** Department of Chemical Engineering, Tokyo University of Agriculture and Technology, Tokyo 184-8588, Japan

**Keywords:** thermosensitive hydrogel, shape-memory function, crystalline-to-amorphous transition, stearyl acrylate, biocompatible polymer

## Abstract

Stimuli-sensitive hydrogels are highly desirable candidates for application in intelligent biomaterials. Thus, a novel thermosensitive hydrogel with shape-memory function was developed. Hydrophobic stearyl acrylate (SA), hydrophilic methoxy poly(ethylene glycol) acrylate (MPGA), and a crosslinking monomer were copolymerized to prepare poly(SA-*co*-MPGA) gels with various mole fractions of SA (*x*_SA_) in ethanol. Subsequently, the prepared gels were washed, dried, and re-swelled in water at 50 °C. Differential scanning calorimetric (DSC) and compression tests at different temperatures revealed that poly(SA-*co*-MPGA) hydrogels with *x*_SA_ > 0.5 induce a crystalline-to-amorphous transition, which is a hard-to-soft transition at ~40 °C that is based on the formation/non-formation of a crystalline structure containing stearyl side chains. The hydrogels stored in water maintained an almost constant volume, independent of the temperature. The poly(SA-*co*-MPGA) hydrogel was soft, flexible, and deformed at 50 °C. However, the hydrogel stiffened when cooled to room temperature, and the deformation was reversible. The shape-memory function of poly(SA-*co*-MPGA) hydrogels is proposed for potential use in biomaterials; this is partially attributed to the use of MPGA, which consists of relatively biocompatible poly(ethylene glycol).

## 1. Introduction

Stimuli-sensitive hydrogels are promising candidates for use in soft actuators and intelligent biomaterials. Hydrogel actuators based on stimuli-sensitive polymers can alter their shape, size, or strength in response to external stimuli, such as heat, pH, light, and magnetic fields, resulting in flexible, complex mechanical motion and shape-memory function [1,2,3]. Robust mechanical and highly flexible properties are required for biomaterials such as artificial muscles, tendons, and ligaments. Hydrogels with excellent properties include slide-ring [4], double network [5], and tri-branched hydrogels [6].

Poly (*N*-isopropylacrylamide) (poly(NIPA)) is a polymer that is extensively used in hydrogel actuators. Poly(NIPA) is thermosensitive, with a lower critical solution temperature of ~33 °C in water [7,8]. Additionally, poly(NIPA) exhibits a hydrophilic/hydrophobic transition in response to temperature variation, and its hydrogel induces a volume phase transition. Thus, poly(NIPA)-based hydrogel actuators can stretch, shrink, bend, and twist [9,10,11,12,13]. However, its poor mechanical strength and thermosensitive volumetric changes may be disadvantageous for certain applications.

Poly(stearyl acrylate) (poly(SA)) is a thermosensitive polymer. Hydrophobic poly(SA) absorbs lipophilic solvents, but not water, and forms an organogel [14] instead of a hydrogel. Hydrogels consisting of poly(SA) were prepared by copolymerization of SA with a hydrophilic monomer. In the 1990s, Osada et al. [15,16,17] developed poly(SA-*co*-AA) (AA: acrylic acid) hydrogels that induced a crystalline-to-amorphous transition, which is an order-disorder transition associated with interactions between alkyl side chains at ~40 °C (depending on the monomer composition). This resulted in a significant change in the Young’s modulus and shape-memory function of the material. Furukawa et al. [18,19,20,21] developed poly(SA-*co*-DMAA) (DMAA: *N*,*N*-dimethylacrylamide) hydrogels for applications such as artificial lenses, bandages, and three-/four-dimensional (3D/4D) printing. Additionally, poly(SA-*co*-AM) (AM: acrylamide) was developed [22]. Thus, poly(SA)-based hydrogels induce a hard-to-soft transition in response to temperature while maintaining a constant volume.

**Figure 1:**
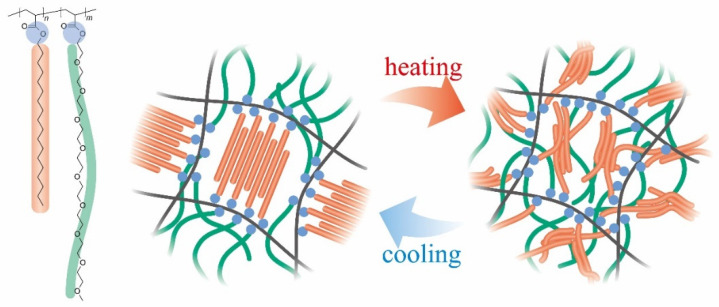
Chemical structure of poly(SA-*co*-MPGA) and an illustration of the crystalline-to-amorphous transition.

In this study, the development and characterization of a novel poly(SA-*co*-MPGA) (MPGA: methoxy poly(ethylene glycol) acrylate) hydrogel as a potential thermosensitive biomaterial is reported. Notably, poly(ethylene glycol) (PEG) is a more biocompatible material than AA, AM, and DMAA; therefore, the poly(SA-*co*-MPGA) hydrogel is suitable for biomedical applications. Differential scanning calorimetric (DSC), compression, and shape-memory tests are conducted in relation to the crystalline-to-amorphous transition, as shown in Figure 1.

## 2. Results and Discussion

**Figure 2:**
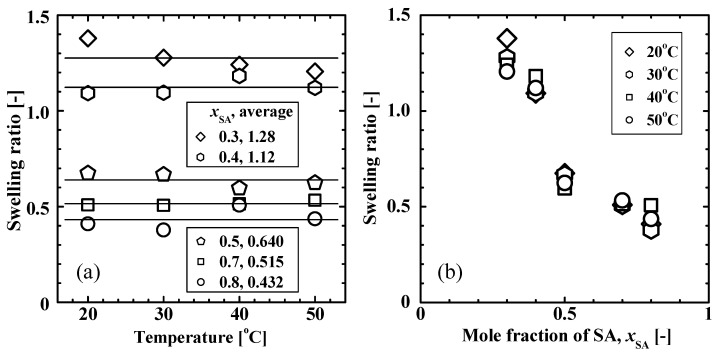
Swelling ratio of poly(SA-*co*-MPGA) hydrogels at 20–50 °C as a function of (**a**) temperature and (**b**) the mole fraction of SA, *x*_SA_, in the pre-gel solution. The solid horizontal lines in (**a**) show the average swelling ratio at 20–50 °C.

Figure 2 shows the swelling ratio of poly(SA-*co*-MPGA) hydrogels at 20–50 °C as a function of (a) temperature and (b) the mole fraction of SA, *x*_SA_, in the pre-gel solution. Figure 2a shows the average values of the swelling ratio (that is, the size) of the hydrogels in the temperature range of 20–50 °C. The swelling ratio was almost constant and independent of the temperature. The swelling ratio increased with a decrease in *x*_SA_, which corresponds to an increase in the mole fraction of hydrophilic MPGA. The hydrogels with *x*_SA_ <0.4 had a swelling ratio of >1, indicating a good water-swollen state. Hydrogels with *x*_SA_ >0.5 induced the crystalline-to-amorphous transition, as described later, and had a swelling ratio of <1.

The *W*/*W*_dry_ ratio was determined as a measure of the water absorption capacity of the gel, where *W* and *W*_dry_ were the masses of the hydrogel and dry gel, respectively. The *W*/*W*_dry_ for poly(SA-*co*-MPGA) with *x*_SA_ = 0.5 was 2.94 at 50 °C. For reference, the *W*/*W*_dry_ values reported in the literature were ~1.4 for poly(SA-*co*-DMAA) [21] and ~4.5 for poly(SA-*co*-AM) [22].

**Figure 3:**
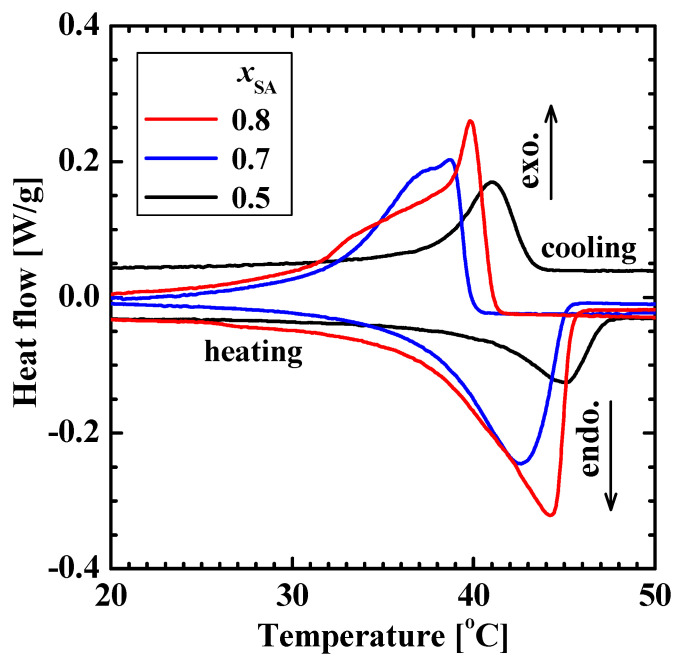
DSC thermograms of poly(SA-*co*-MPGA) hydrogels prepared with *x*_SA_ = 0.5, 0.7, and 0.8.

Figure 3 shows the DSC thermograms of the poly(SA-*co*-MPGA) hydrogels prepared with *x*_SA_ = 0.5, 0.7, and 0.8. These hydrogels had endothermic and exothermic peaks, whereas the hydrogel prepared with *x*_SA_ = 0.3 did not exhibit these peaks. A similar DSC thermogram for poly(SA) was reported in the literature [14,21]. Poly(SA) induces a crystalline-to-amorphous transition; the hydrophobic stearyl side chains form a crystalline structure at temperatures below the crystallization temperature *T*_c_, and their packing becomes amorphous at temperatures above the melting temperature *T*_m_. Previously, the *T*_m_ and *T*_c_ of the dry poly(SA) gel were reported to be 44.8 and 41.8 °C, respectively [14]. The DSC results demonstrated that the poly(SA-*co*-MPGA) hydrogel also induced a crystalline-to-amorphous transition (as shown in Figure 1). The *T*_c_ values were 43.3, 39.8, and 41.1 °C for poly(SA-*co*-MPGA) hydrogels prepared with *x*_SA_ = 0.5, 0.7, and 0.8, respectively. The *T*_m_ value was slightly unclear owing to the broad DSC peak, which was slightly higher than the *T*_c_. The peak area for the poly(SA-*co*-MPGA) hydrogel prepared with *x*_SA_ = 0.5 was smaller than that of the hydrogels prepared with *x*_SA_ = 0.7 and 0.8, based on the amount of SA units per gram of hydrogel.

**Figure 4:**
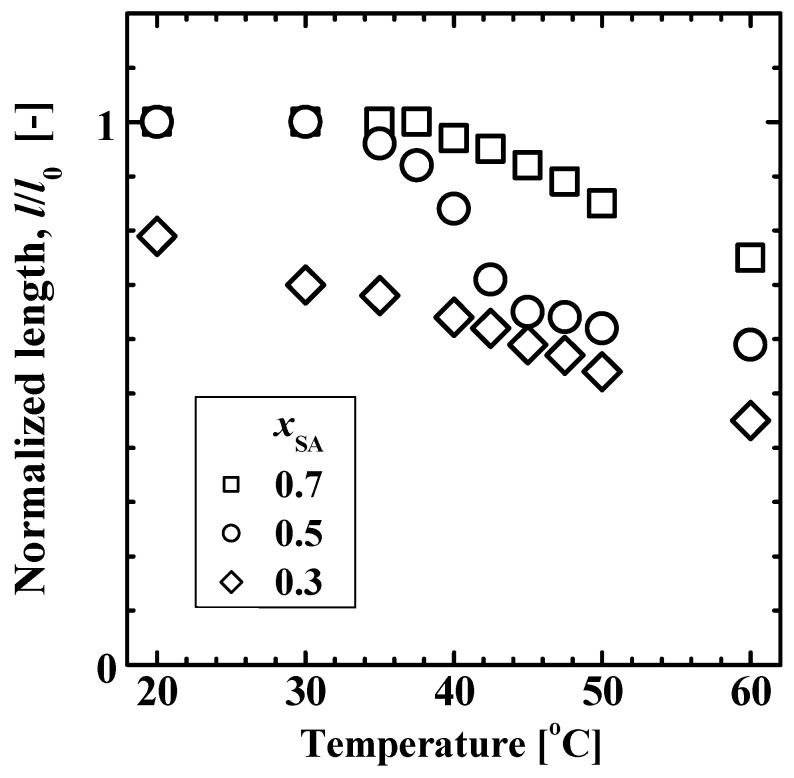
Normalized length, *l*/*l*_0_, of cylinder-shaped poly(SA-*co*-MPGA) hydrogels with *x*_SA_ = 0.3, 0.5, and 0.7 as a function of temperature. *l*_0_ is the initial length at 20 °C. *l* is the hydrogel length loaded with weight (36 g) at a given temperature.

Figure 4 shows the compression test results of the cylindrical poly(SA-*co*-MPGA) hydrogels using a weight (36 g) as a load under various temperature conditions. The normalized length *l*/*l*_0_ of the hydrogel prepared with *x*_SA_ = 0.7 was ~1 at 20–37.5 °C, and it decreased with an increase in temperature (>40 °C). This behavior indicates that the hydrogel stiffened <37.5 °C and softened >40 °C. The change in the hardness and softness, that is, the hard-to-soft transition, of the hydrogel was attributed to the crystalline-to-amorphous transition of the stearyl side chains of SA. The crystalline structure of the stearyl side chains function as pseudo-crosslinking points (as shown in Figure 1), enhancing the hydrogel strength. The hydrogel prepared with *x*_SA_ = 0.5 exhibited a similar thermosensitive behavior; however, the *l*/*l*_0_ value was smaller at temperatures of >40 °C. The hydrogel prepared with *x*_SA_ = 0.3 had an *l*/*l*_0_ value of ~0.8 at 20 °C, confirming its softness.

The hydrogel strength at temperatures of >40 °C decreased with a decreasing *x*_SA_. The hydrogel strength is primarily influenced by the swelling ratio shown in Figure 2. The rubber network theory, which was derived based on the statistical mechanics of crosslinked polymer networks, describes the relationship *τ* ∝ (*ν*_e_ *ϕ*_p_^–2/3^) in the stress-strain curves of the tensile or compressive strengths of gels, where *τ* is the stress required for a given deformation, *ν*_e_ is the effective crosslinking density, and *ϕ*_p_ is the volume fraction of the polymer in the hydrogel [23,24,25]. As shown in Figure 2, a decrease in *x*_SA_ causes an increase in the swelling ratio, resulting in reductions in *ν*_e_ and *ϕ*_p_ and consequently a decrease in *τ*.

**Figure 5:**
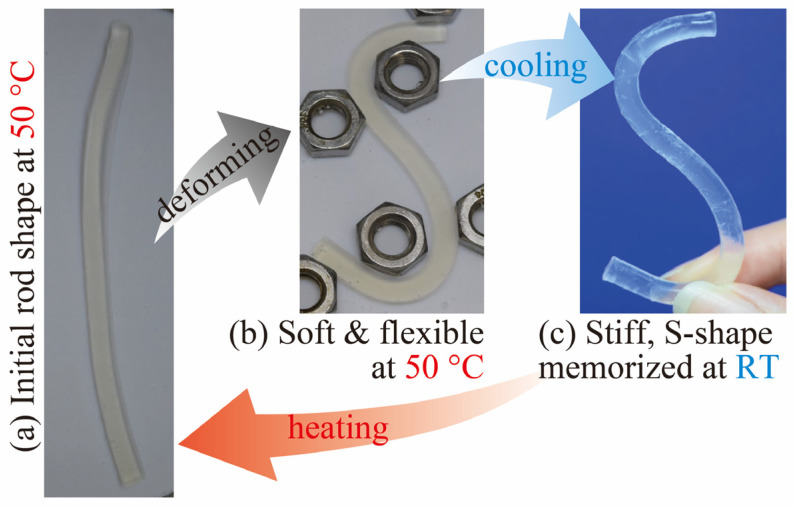
Photographs of the shape-memory function of poly(SA-*co*-MPGA) hydrogel with *x*_SA_ = 0.5: (**a**,**b**) in water at 50 °C and (**c**) at room temperature (~20 °C).

Figure 5 shows the shape memory function of the poly(SA-*co*-MPGA) hydrogel prepared with *x*_SA_ = 0.5. Initially, the hydrogel was rod-shaped. The hydrogel was soft and flexible at 50 °C and deformed into an S shape. When the hydrogel was cooled to room temperature, it stiffened, and the S shape was fixed. When the hydrogel was heated to 50 °C, it reverted to its original shape. The deformation based on the hard-to-soft transition induced by heating or cooling occurs within a few minutes and repeatedly. The S-shaped hydrogel structure can be retained indefinitely in water at room temperature, which was confirmed for several months.

The poly(SA-*co*-MPGA) hydrogel deforms at a temperature slightly higher than body temperature and stiffens at body temperature. Thus, the poly(SA-*co*-MPGA) hydrogel is proposed for use as a cast-like, anti-adhesive, or stent material with a well-fitted shape to reinforce and protect injured or post-surgery organs and tissues in the body.

## 3. Conclusions

Poly(SA-*co*-MPGA) hydrogels were prepared by free-radical copolymerization of SA, MPGA, and EGDM in ethanol, followed by washing, drying, and re-swelling in water at 50 °C. The DSC and compression tests performed at different temperature conditions revealed that poly(SA-*co*-MPGA) hydrogels with *x*_SA_ >0.5 induce a crystalline-to-amorphous transition, which is a hard-to-soft transition that occurs at ~40 °C. The hydrogels had an almost constant volume, independently of the temperature. The shape-memory function of poly(SA-*co*-MPGA) hydrogel is that it is soft, flexible, and deformed at temperatures of >40 °C and that it stiffens when cooled to <37.5 °C. Additionally, the deformation of hydrogel is reversible.

## 4. Materials and Methods

### 4.1. Preparation of Poly(SA-co-MPGA) Gels

Copolymer gels with various concentrations of SA and MPGA (average molecular weight: 483) were synthesized by free-radical polymerization. Ethanol was used as a solvent to dissolve hydrophobic SA and hydrophilic MPGA. The monomer solution contained SA, MPGA, ethylene glycol dimethacrylate (EGDM; crosslinking monomer), and *N*,*N*,*N*′,*N*′-tetramethylethylenediamine (TEMED; polymerization accelerator). The initiator solution contained 2,2′-azobis(2,4-dimetylvaleronitrile) (ADVN; polymerization initiator). Nitrogen gas was bubbled through each solution for 1 h to remove dissolved oxygen. Subsequently, the initiator solution was added to the monomer solution in a polytetrafluoroethylene (PTFE) tube (inner diameter: 6 mm). Polymerization was performed at 60 °C for 1 d in a nitrogen atmosphere. The overall concentration of the primary monomers in the pre-gel solution was 1000 mol/m^3^; for example, 700 mol/m^3^ of SA and 300 mol/m^3^ of MPGA, corresponding to an SA mole fraction, *x*_SA_, of 0.7. The concentrations of EGDM, TEMED, and ADVN were 100, 30, and 20 mol/m^3^, respectively, for all the gels. The resulting gels were cut into cylinders with a length of 6 mm. Subsequently, the gels were washed with ethanol at 50 °C to remove non-crosslinked chemicals and then dried in an oven at 50 °C.

### 4.2. Swelling Properties in Water

The dry, cylindrical gel was immersed in water at 50 °C for several days, and water was absorbed to obtain the poly(SA-*co*-MPGA) hydrogel. The hydrogel diameter, *d* [mm], at swelling equilibrium was measured using a photograph taken with a digital camera. Subsequently, the hydrogel diameter was measured at 40, 30, and 20 °C. The swelling ratio was defined as the hydrogel volume divided by the volume of the as-synthesized gel, and was calculated as follows: (*d*/6)^3^.

### 4.3. Compression Test

The temperature dependence of the softness and hardness of the poly(SA-*co*-MPGA) hydrogel was evaluated. The cylindrical hydrogel was vertically placed in a glass test tube, and water was added to half the height of the hydrogel. Subsequently, the test tube was placed in a constant-temperature water bath at 20 °C. The initial length l0 of the hydrogel at 20 °C was measured using a digital camera. A total weight (36 g) was placed on the hydrogel. Subsequently, the length l of the hydrogel was measured after several minutes. Under a continuous load, the temperature was increased stepwise, and the hydrogel length was measured at each temperature.

### 4.4. DSC Analysis

A differential scanning calorimeter (DSC-60, Shimadzu Co., Kyoto, Japan) was used to perform DSC analysis. The poly (SA-*co*-MPGA) hydrogel was ground, and the ground sample (3.2 mg) was enclosed in an aluminum cell. α-Alumina was used as a reference material and enclosed in another cell. The cells were placed in a sample chamber under nitrogen gas flow. For DSC measurements, the cells were heated and subsequently cooled between 0 and 60 °C at a rate of 2 °C/min.

## Data Availability

Not applicable.

## References

[1] Apsite I., Salehi S., Ionov L. (2022). Materials for smart soft actuator systems. Chem. Rev..

[2] Kim J., Kim J.W., Kim H.C., Zhai L., Ko H.U., Muthoka R.M. (2019). Review of soft actuator materials. Int. J. Precis. Eng. Manuf..

[3] Han I.K., Chung T., Han J., Kim Y.S. (2019). Nanocomposite hydrogel actuators hybridized with various dimensional nanomaterials for stimuli responsiveness enhancement. Nano Converg..

[4] Liu C., Morimoto N., Jiang L., Kawahara S., Noritomi T., Yokoyama H., Mayumi K., Ito K. (2021). Tough hydrogels with rapid self-reinforcement. Science.

[5] Nonoyama T., Gong J.P. (2021). Tough double network hydrogel and its biomedical applications. Annu. Rev. Chem. Biomol. Eng..

[6] Fujiyabu T., Sakumichi N., Katashima T., Liu C., Mayumi K., Chung U.I., Sakai T. (2022). Tri-branched gels: Rubbery materials with the lowest branching factor approach the ideal elastic limit. Sci. Adv..

[7] Hirokawa Y., Tanaka T. (1984). Volume phase transition in a nonionic gel. J. Chem. Phys..

[8] Tokuyama H., Mori H., Hamaguchi R., Kato G. (2021). Prediction of the lower critical solution temperature of poly(*N*-isopropylacrylamide-co-methoxy triethyleneglycol acrylate) in aqueous salt solutions using support vector regression. Chem. Eng. Sci..

[9] Deng K., Rohn M., Gerlach G. (2016). Design, simulation and characterization of hydrogel-based thermal actuators. Sens. Actuators B.

[10] Warren H., Shepherd D.J., in het Panhuis M., Officer D.L., Spinks G.M. (2020). Porous PNIPAm hydrogels: Overcoming diffusion-governed hydrogel actuation. Sens. Actuators A.

[11] Choi J.G., Spinks G.M., Kim S.J. (2022). Mode shifting shape memory polymer and hydrogel composite fiber actuators for soft robots. Sens. Actuators A.

[12] Liu J., Jiang L., Liu A., He S., Shao W. (2022). Ultrafast thermo-responsive bilayer hydrogel actuator assisted by hydrogel microspheres. Sens. Actuators B.

[13] Tokuyama H., Sasaki M., Sakohara S. (2006). Preparation of a novel composition-gradient thermosensitive gel. Colloids Surf. A.

[14] Tokuyama H., Kato Y. (2010). Preparation of thermosensitive polymeric organogels and their drug release behaviors. Eur. Polym. J..

[15] Matsuda A., Sato J., Yasunaga H., Osada Y. (1994). Order-disorder transition of a hydrogel containing an *N*-alkyl acrylate. Macromolecules.

[16] Osada Y., Matsuda A. (1995). Shape memory in hydrogels. Nature.

[17] Kagami Y., Gong J.P., Osada Y. (1996). Shape memory behaviors of crosslinked copolymers containing stearyl acrylate. Macromol. Rapid Commun..

[18] Hasnat Kabir M., Gong J., Watanabe Y., Makino M., Furukawa H. (2013). Hard-to-soft transition of transparent shape memory gels and the first observation of their critical temperature studied with scanning microscopic light scattering. Mater. Lett..

[19] Hasnat Kabir M., Hazama T., Watanabe Y., Gong J., Murase K., Sunada T., Furukawa H. (2014). Smart hydrogel with shape memory for biomedical applications. J. Taiwan Inst. Chem. Eng..

[20] Shiblee M.D.N.I., Ahmed K., Yamazaki Y., Kawakami M., Furukawa H. (2021). Light scattering and rheological studies of 3D/4D printable shape memory gels based on poly (*N*,*N*-dimethylacrylamide-*co*-stearyl acrylate and/or lauryl acrylates). Polymers.

[21] Kabir M.H., Ahmed K., Furukawa H. (2016). The effect of cross-linker concentration on the physical properties of poly(dimethyl acrylamide-*co*-stearyl acrylate)-based shape memory hydrogels. Microelectron. Eng..

[22] Lin X.K., Chen L., Zhao Y.P., Dong Z.Z. (2010). Synthesis and characterization of thermoresponsive shape-memory poly(stearyl acrylate-*co*-acrylamide) hydrogels. J. Mater. Sci..

[23] Flory P.J., Rehner J. (1943). Statistical mechanics of cross–linked polymer networks II. Swelling. J. Chem. Phys..

[24] James H.M., Guth E. (1943). Theory of the elastic properties of rubber. J. Chem. Phys..

[25] Tokuyama H., Nakahata Y., Ban T. (2020). Diffusion coefficient of solute in heterogeneous and macroporous hydrogels and its correlation with the effective crosslinking density. J. Membr. Sci..

